# Clinicians’ Attitudes Towards Do-Not-Resuscitate Directives in a Teaching Hospital in Saudi Arabia

**DOI:** 10.7759/cureus.6510

**Published:** 2019-12-30

**Authors:** Mohammed Madadin, Gada M Alsaffar, Sara M AlEssa, Afnan Khan, Dania A Badghaish, Shahad M Algarni, Ritesh G Menezes

**Affiliations:** 1 Department of Pathology, College of Medicine, Imam Abdulrahman Bin Faisal University, Dammam, SAU; 2 Department of Internal Medicine, College of Medicine, Imam Abdulrahman Bin Faisal University, Dammam, SAU

**Keywords:** do not resuscitate, intensive care unit, decision making, cardiopulmonary resuscitation, saudi arabia, dnr rate, king fahd hospital of the university

## Abstract

The Do-Not-Resuscitate (DNR) directive has provided a major leap in end-of-life care. To demonstrate the factors influencing physicians’ DNR decisions in King Fahd University Hospital in the Eastern Province of Saudi Arabia, 42 physicians from the medical and surgical departments of the same center were requested to participate in a cross-sectional survey.

Thirty-six questionnaires were completed and returned from a total of 42 distributed among physicians, making a response rate of 85.7%. Certain diagnostic categories increase the likelihood of issuing a DNR order for a patient. Neurological (58.3%) and cardiovascular (41.7%) diseases were the highest response among other diseases in influencing physicians’ decisions. In addition, other factors like lack of comorbidities (55.5%), age (52.7%), and previous intensive care unit (ICU) admissions and resuscitation (44.4%) showed an effect on the directive decisions of DNR among investigated physicians. However, weak palliative care in the hospital (11.1%), religious beliefs (5.5%), and gender (2.7%) were the least associated factors affecting physicians’ DNR decisions.

This study addresses the influencing factors of DNR orders issuance among King Fahd Hospital of the University physicians. Physicians noted that cultural standards and religious beliefs do play a role in their decision-making but had less of an effect as compared to other clinical data such as comorbidities, age, and previous ICU admissions.

## Introduction

Before the introduction of Do-Not-Resuscitate (DNR) in the 1970s, cardiopulmonary resuscitation (CPR) was performed on every patient with cardiopulmonary arrest, regardless of their clinical situations and prognoses. This had inevitably resulted in unfavorable results, including the prolongation of patients suffering from terminal illnesses and increased family stress in the form of anxiety and financial burden [1].

DNR is a medical directive to withhold the patients’ cardiac and pulmonary resuscitation. It was initially passed by the fatwa (Islamic ruling) issued on 30/6/1409 (1988/1989), No. 12086. It states that DNR is a medical decision agreed upon by three competent physicians when the treatment is deemed futile in terminally ill patients [2]. Moreover, the fatwa in Saudi Arabia specifies that the decision is to be solely made by the physicians and that families are not qualified to make this decision. However, the reasoning behind it should be clearly explained to the patient and family [3]. The latest Saudi National Policy of 2017 adds some specifics to be followed but has not altered that the decision is to be carried out by the same number of physicians [2].

Before the development of clear guidelines in Saudi Arabia, a study was done to examine physicians’ practice and attitude towards DNR orders; 70% of them believed that it was the physician’s medical decision to take, with attitudes ranging similarly between paternalistic, passive, and balanced [4]. Al-Sheef reported that in the outpatient setting, a third of their sample believed DNR to be against Islamic beliefs, but the majority showed a willingness to learn more about the National Policy in Saudi Arabia [3]. However, a study conducted in the Western Province showed that around 96% of the emergency and intensive care unit (ICU) physicians believed DNR to be consistent with Islamic beliefs, and the majority of them considered DNR in terminal illnesses [5].

Furthermore, Gouda et al. evaluated the understanding and adherence of physicians to the DNR policy in their center. They reported shortcomings in patient and family explanations and delays in form completion [5-6]. This clarifies the dissatisfaction of families and may confound the effect of the families’ cultural beliefs [5-6].

A questionnaire has been distributed among physicians treating patients in the ICUs. The questionnaire was designed in an attempt to assess the major domains of physician identification, knowledge, attitude, and practice of the DNR directive. Reflecting the practice by the questionnaire ultimately establishes the defining elements of DNR decision-making.

From our literature review, we note the significant gaps in data collection within Saudi Arabia, especially in the Eastern Province, and, therefore, what has been previously reported cannot be generalized. We intend to gather sufficient data and hopefully reach a full understanding of DNR dynamics within Saudi Arabia, from patients to physicians.

## Materials and methods

Subjects

A cross-sectional approach was implemented in the study. To analyze the factors influencing physicians in DNR order decision-making, a structured self-administered questionnaire was distributed to physicians in the medical and surgical ICUs in King Fahd Hospital of the University (KFHU). A total of 42 physicians were included in the sample; of those, 36 answered questionnaires were collected.

The questionnaire construction was influenced by multiple reliable and validated surveys, questionnaires, and assessment tools based on the cited literature. In addition, it has been validated by experts in the respective field and was found to be reliable.

Variables, materials, and procedures

The Acute Physiology and Chronic Health Evaluation (APACHE) II is a commonly used classification system in ICUs for the measurement of illness severity [7]. Its original 50 diagnostic categories were collapsed into six main categories: respiratory, cardiovascular, neurological, gastrointestinal, polytrauma, and others.

To analyze the factors influencing physicians in DNR order decision-making, a structured questionnaire was distributed. Several categories of questions were organized to assess the major domains of physician identification, knowledge, attitude, and practice of the DNR directive; these included physician traits, experience, and personal beliefs; legal and authority issues; patients’ medical conditions and severity; cultural inclination and religious beliefs; and the effect of the decision on future care.

Data management and analysis

The Statistical Package for Social Sciences (SPSS version 223, SPSS Inc, Chicago, IL) was used for data analysis. The P-value of significance was taken to be < 0.05. The data were analyzed descriptively using either frequency and percentages or mean and standard deviation. A bar chart was used to graphically compare the data.

## Results

Physician information

A total of 42 questionnaires were distributed among physicians, with 36 being completed and returned, making the response rate 85.7%. All included physicians, from various medical and surgical specialties, have dealt with patients in the ICU, with the majority being consultants (88.9%). Fifty-one point four percent of the physicians’ ICU experience ranged from zero to five years, 34.3% of the physicians had an experience of six to 19 years, and only 14.3% had 20 or more years of experience. The demographics of the physicians revealed that 52.8% of them were Saudi, 86.1% were Muslim, and 77.8% were male.

Knowledge of authority and legal issues

All of the participants agreed that DNR is legal in Saudi Arabia, however, 83.3% believed it to be allowed in Islam, 2.8% thought it was prohibited in Islam, and 13.9% did not know. Ninety-one point seven percent were aware of the presence of a DNR policy in KFHU, and 67.7% knew about Saudi Arabia’s “National Policy and Procedure for DNR status.” Fifty-two point eight percent knew that the patient’s and/or family’s consent was not required to issue a DNR order, and 91.4% believed that DNR status can be issued to any age. Physician understanding of those eligible to participate in the decision are as follows: 44% of the physicians thought that an intensivist must participate in the decision, and 58% agreed that the treating physician must be part of the decision team, with 36% correctly stating that any competent physician can participate.

Experience

Of the total respondents, 86.1% had previously issued a DNR order, with 96.8% of those reporting their DNR orders were 0%-10% of their patients, over the past year. Of the participants, 41.6% stated that they discussed DNR with ≤ 10% of the patients or families regardless of the outcome while 44.4% discussed DNR status with 76%-100% of the patients or families.

Physician comfort in discussing DNR with the patient or family ranged between very comfortable, somewhat comfortable, and not comfortable, with very similar rates. Seventy-seven point eight percent stated that they do not hesitate to approach families or patients with the subject of DNR. The majority (75%) of the participants preferred early decisions regarding DNR status (before the last three days of life), however, 50% noted that they prefer to discuss this decision with the patient or family at signs of deterioration.

Sixty-six point seven percent of the physicians declared that the family’s refusal did not deter them from the DNR decision, however, of those, 41.7% may hesitate but still go through with the decision. Forty-four point four percent stated that the most difficult age group to issue a DNR for is 18-35 years old while 50% of the respondents stated that age does not affect their decision. The majority (80.6%) of the physicians agreed that they are more likely to issue a DNR for patients with multiple comorbidities, and 77.8% agreed that oncological patients are also more likely to have DNR code status.

Various opinions were answered regarding the change in the quality of care once a DNR order is issued; 30.6% strongly disagreeing and only 5.6% strongly agreeing. Finally, 69.4% agreed that more palliative care is given when a DNR order is issued.

**Figure 1:**
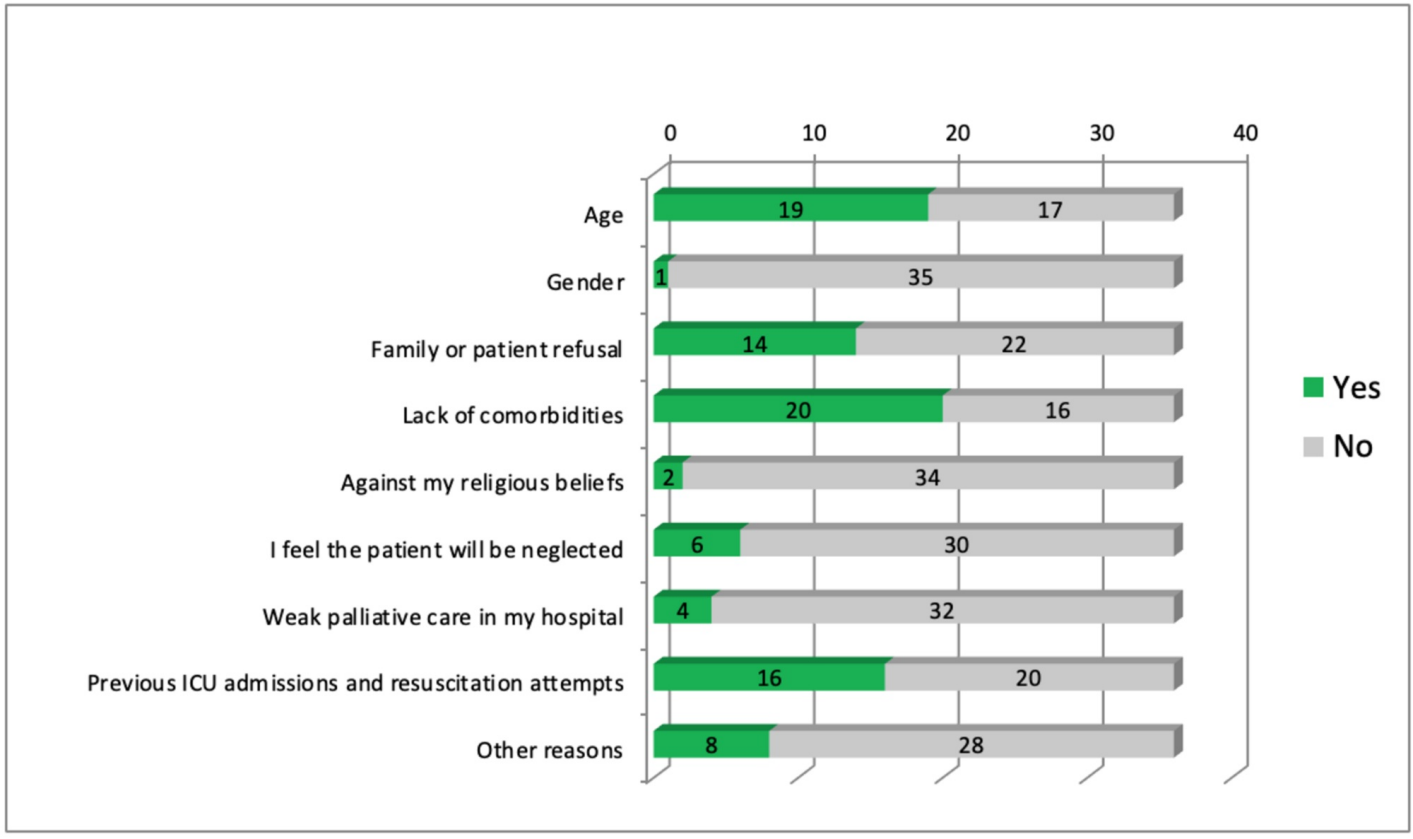
Factors influencing physician’s decision in issuing the DNR order DNR: Do-Not-Resuscitate

Table 1 shows the participants’ responses to whether certain diagnostic categories increase the likelihood of issuing a DNR order for a patient. Neurological and cardiovascular disease being the most popular answers: 58.3%, and 41.7%, respectively. Figure 1 explores other factors that may influence the physician’s decision.

**Table 1:** Diagnostic categories associated with a higher likelihood of DNR: participants DNR: Do-Not-Resuscitate

Diagnostic Category	Frequency
Cardiovascular diseases	
Yes	15 (41.7%)
No	21 (58.3%)
Pulmonary diseases	
Yes	8 (22.2%)
No	28 (77.8%)
Neurological diseases	
Yes	21 (58.3%)
No	15 (41.7%)
Gastrointestinal diseases	
Yes	3 (8.3%)
No	33 (91.7%)
Other diseases	
Yes	16 (44.4%)
No	20 (55.6%)

## Discussion

Do-Not-Resuscitate (DNR) is a medical order that directs towards withholding resuscitation in cases of cardiac and pulmonary arrest. It is employed in clinical settings where further treatment may only prolong patient and family suffering without the anticipated benefit [3]. Moreover, the Islamic Medical Association of North America states that this preserves dignity when death is imminent [8]. The concept of a dignified death is widely shared throughout other cultures such as the Japanese [9].

Physicians’ knowledge

All respondents were cognizant that DNR in Saudi Arabia is legal, which demonstrates a widespread knowledge of this law. However, only 83.3% of them knew that it was allowed in Islam. This sheds light on a possible misconception regarding the fatwa and, subsequently, indicates a knowledge gap regarding Saudi Arabia’s constitution; which is Islamic law. Moreover, of the participants, 3.1% were non-Muslim and were not expected to have prior knowledge of the Islamic fatwa.

The DNR policy in KFHU states that it is a medical decision of three competent physicians and patient and/or family consent is not required. Among the participants, 91.7% were aware of the presence of a DNR policy in our hospital. Yet, 52.1% knew that families and patients’ consent was not required and 91.4% knew that DNR can be issued for all ages.

Likewise, Gouda demonstrated that most of their physician sample knew about the presence of a DNR policy in their institution but were not familiar with its specifics [5]. This guides us in recognizing the nuances between having a policy set and its execution.

Patient communication

The National Policy’s procedures include communicating the decision to the family or patients and clarifying that it is purely a medical decision [2]. Moreover, the American Heart Association recommended that when deciding against CPR, it should be widely communicated throughout the staff [1].

Fifty percent of the respondents discussed DNR status with patients or families at signs of deterioration. Likewise, in serious illnesses, such as cancer, the physicians were more likely to discuss DNR orders when the prognosis was poor [9]. Twenty-two point two percent of the physicians reported that they hesitate to approach the subject, which could explain the delayed communication to the patients and families, as observed by Connors et al. [10].

Diagnostic category

According to the physicians’ responses, diagnostic categories, in general, did not have a statistical implication on issuing DNR orders. However, neurocritical physicians reported that they provided DNR orders to approximately 26% to 50% of their patients. In addition, 58.3% of the respondents agreed that neurological conditions are more likely to warrant DNR status compared to other clinical entities. The inclusion of the debilitating nature of these diseases, neurological malignancies, inoperable situations, decreased mental competence, and comatose states all factor in the higher prevalence of DNR [11-12]. Moreover, the majority agreed that cancer patients are more likely to get DNR. Huang et al. noted that 80% of their patient sample had a DNR order, of which they were all terminally ill with cancer [13].

Culture

Culture plays an important role in the physicians’ decision; it appeared that those dealing with terminal or severely ill patients regularly were more open to exploring the benefits of DNR and had an accurate understanding of it. However, those who are not, or were older, were more likely to view the subject as taboo, misinterpreting it as an order to withdraw life support rather than merely life-sustaining measures. Saeed et al. reported in their study that cultural beliefs and countries of practice play a crucial role in the outcome of the health care worker’s decision [14].

In Taiwan, Lin recounted that certain religious groups have different opinions concerning DNR decisions; Buddhism and Daoism being more likely to refuse DNR [15]. Another study described Christians, Muslims, and Jews as more willing to agree to advance directives such as DNR [16].

## Conclusions

This study addresses the influencing factors of DNR orders issuance among KFHU physicians. Physicians noted that cultural standards and religious beliefs do play a role in their decision-making but had less of an effect as compared to other clinical data, such as comorbidities, age, and previous ICU admissions. Our research was conducted in a single-center, university-affiliated secondary hospital. A multicenter study would be more explanatory in this regard. The possibility of selection bias cannot be excluded, as some physicians were reluctant to fill out the questionnaire for the topic being highly sensitive for them. For a decision as subjective as DNR utilization, it is challenging to justify for all the confounders that may account in the decision making.

We have found a scarcity of DNR-related research in the Arab countries that may be attributed to cultural matters. Emphasizing its legality in Islam by responsible authorities would break the ice and make it less of a controversy. Also, further studies should be done to assess the practice of DNR in institutes with a greater prevalence of terminal illnesses among patients and to compare them to institutes in which DNR is rarely or never issued.
